# Pemphigus Vulgaris Possibly Associated with COVID-19 Infection

**DOI:** 10.7759/cureus.33897

**Published:** 2023-01-17

**Authors:** Yul Hee Kim, Hee Young Kang

**Affiliations:** 1 Dermatology, Ajou University School of Medicine, Suwon, KOR

**Keywords:** autoimmune bullous disease, keratinocyte, sars-cov-2 spike protein, pemphigus vulgaris, covid-19

## Abstract

Pemphigus vulgaris (PV) represents damage to epidermal keratinocytes, resulting in acantholysis due to the production of autoantibodies against desmoglein-1 and desmoglein-3. Autoimmune blistering disorders such as pemphigus vulgaris or bullous pemphigoid that develop following coronavirus disease 2019 (COVID-19) have been reported in several studies. Herein, we report a case of PV onset following COVID-19 infection in a 17-year-old female, demonstrating ­­the potential pathogenic capacity of SARS-CoV-2 to develop PV.

## Introduction

During the ongoing coronavirus disease 19 (COVID-19) pandemic, multiple skin manifestations have been found after Severe acute respiratory syndrome coronavirus 2 (SARS-CoV-2) infection and vaccination. Autoimmune blistering disorders such as pemphigus vulgaris (PV) or bullous pemphigoid that develop following COVID-19 infection have been reported in several studies [1,2]. However, the identification of a possible pathological association between SARS-CoV-2 and autoimmune bullous disease remains elusive. Recently, we encountered a case of PV onset following COVID-19 infection in a 17-year-old female, demonstrating ­­the potential pathogenic capacity of SARS-CoV-2 to develop PV.

## Case presentation

**Figure 1:**
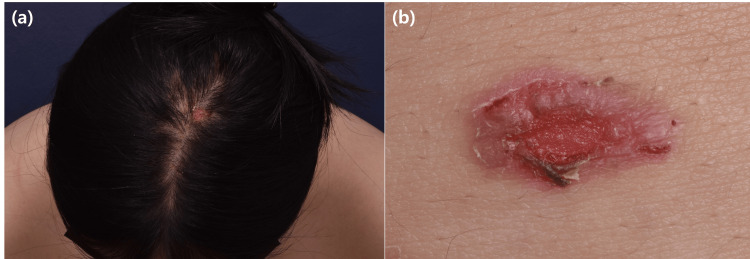
Clinical findings of pemphigus vulgaris after SARS-CoV-2; (a) multiple painful erosive and crusted patches on the scalp and (b) biopsy site from the abdomen. SARS-COV-2: Severe Acute Respiratory Syndrome Coronavirus 2

An otherwise healthy 17-year-old female presented with a six-week history of multiple erosions on the scalp, nose, abdomen, and mucosal area, including the oral and vulva areas (Figure 1). On the history taking, the initial skin lesion was blister, but all of them changed to erosion. A punch biopsy from the abdomen showed suprabasal acantholysis with lymphocytic infiltration, and a direct immunofluorescence finding showing intercellular IgG deposition confirmed a diagnosis of pemphigus vulgaris. The patient reported a COVID-19 infection three days before the skin rash that was confirmed by a nasopharyngeal swab reverse-transcriptase polymerase chain reaction study for SARS-Cov-2 RNA. Because she is under 30 years of age, we were suspicious of COVID-19 triggering PV.

**Figure 2:**
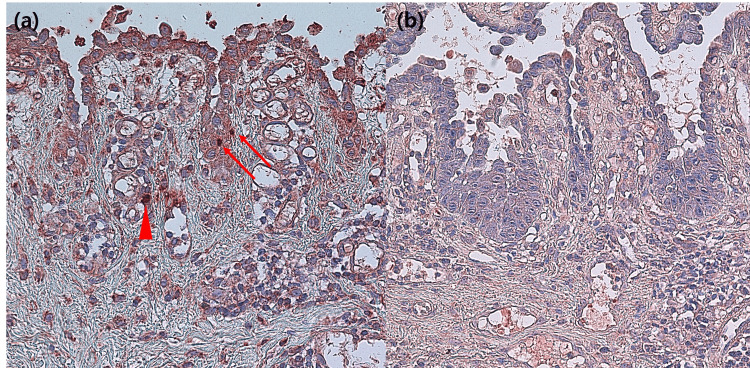
(a) Immunohistochemical study of the SARS-CoV-2 spike protein showing positivity in basal keratinocytes (arrows) and endothelial cells (arrowhead) (original magnification x 400). (b) A representative figure of negative staining of a pemphigus vulgaris specimen before 2019 (original magnification x 400). SARS-CoV-2: Severe Acute Respiratory Syndrome Coronavirus-2

The detection of the SARS-CoV/SARS-CoV-2 spike protein in cutaneous endothelial cells, perivascular lymphocytes, and the epithelial cells of the eccrine epithelium, provides a potential pathogenetic mechanism of COVID-19, especially in cases involving chilblains and pityriasis rosea-like eruptions [3,4]. We, therefore, conducted an immunohistochemical study with an antibody against SARS-CoV/SARS-CoV-2 (dilution 1:300, clone 1A9, GeneTex, Inc., Irvine, CA, USA) [3,4]. The findings showed fine-to-coarse, bright red granular deposits in the nucleus and cytoplasm of the basal keratinocytes as well as in the vascular endothelial cells and epithelial cells of the eccrine glands (Figure 2a). Negative control in the form of three PV specimens before 2019, biopsies from non-COVID-19 related inflammatory dermatoses revealed no staining (Figure 2b). The patient received two doses of rituximab spaced two weeks apart, and the skin lesions slowly healed with periodic follow-ups.

## Discussion

PV represents damage to epidermal keratinocytes, resulting in acantholysis due to the production of autoantibodies against desmoglein-1 and desmoglein-3. Autoimmunity development in PV is a complex process and may be idiopathic or induced by neoplasm, drug, infection, or inflammatory processes [2]. In our study, the SARS-CoV/SARS-CoV-2 spike protein was observed in the basal keratinocytes that suggests a possible pathological link between SARS-CoV-2 and the onset of PV. Interestingly, a recent study showed high expression levels of angiotensin-converting enzyme 2 (ACE2), a cellular receptor of COVID-19, on keratinocytes in human skin and indicated that keratinocytes are potential target cells for the viral infection when a patient has a COVID-19 infection [5]. The limitation of this study is that it was insufficient to confirm the gene expression in our sample.

## Conclusions

In conjunction with this finding, the present study suggests that the presence of the virus in epidermal keratinocytes indicates the pathogenic possibility of PV after SARS-CoV-2, suggesting that SARS-CoV-2 might contribute to the onset of PV by triggering an autoimmune response.

## References

[1] Zou H, Daveluy S (2022). Pemphigus vulgaris after COVID-19 infection and vaccination. J Am Acad Dermatol.

[2] Drenovska K, Vassileva S, Tanev I, Joly P (2021). Impact of COVID-19 on autoimmune blistering diseases. Clin Dermatol.

[3] Santonja C, Heras F, Núñez L, Requena L (2020). COVID-19 chilblain-like lesion: immunohistochemical demonstration of SARS-CoV-2 spike protein in blood vessel endothelium and sweat gland epithelium in a polymerase chain reaction-negative patient. Br J Dermatol.

[4] Welsh E, Cardenas-de la Garza JA, Brussolo-Marroquín E, Cuellar-Barboza A, Franco-Marquez R, Ramos-Montañez G (2022). Negative SARS-CoV-2 antibodies in patients with positive immunohistochemistry for spike protein in pityriasis rosea-like eruptions. J Eur Acad Dermatol Venereol.

[5] Xue X, Mi Z, Wang Z, Pang Z, Liu H, Zhang F (2021). High expression of ACE2 on keratinocytes reveals skin as a potential target for SARS-CoV-2. J Invest Dermatol.

